# Effects and Underlying Mechanisms of Bioactive Compounds on Type 2 Diabetes Mellitus and Alzheimer's Disease

**DOI:** 10.1155/2019/8165707

**Published:** 2019-01-17

**Authors:** Rongzi Li, Yuxian Zhang, Suhail Rasool, Thangiah Geetha, Jeganathan Ramesh Babu

**Affiliations:** Department of Nutrition, Dietetics, and Hospitality Management, Auburn University, Auburn, Alabama 36849, USA

## Abstract

Type 2 diabetes mellitus is a complicated metabolic disorder characterized by hyperglycemia and glucose intolerance. Alzheimer's disease is a progressive brain disorder characterized by a chronic loss of cognitive and behavioral function. Considering the shared characteristics of both diseases, common therapeutic and preventive agents may be effective. Bioactive compounds such as polyphenols, vitamins, and carotenoids found in vegetables and fruits can have antioxidant and anti-inflammatory effects. These effects make them suitable candidates for the prevention or treatment of diabetes and Alzheimer's disease. Increasing evidence from cell or animal models suggest that bioactive compounds may have direct effects on decreasing hyperglycemia, enhancing insulin secretion, and preventing formation of amyloid plaques. The possible underlying molecular mechanisms are described in this review. More studies are needed to establish the clinical effects of bioactive compounds.

## 1. Introduction

Diabetes is a complex metabolic disorder that is characterized by hyperglycemia due to insulin insufficiency and/or insulin dysfunction. Globally, an estimated 425 million adults were living with diabetes mellitus in 2017. By 2045, projections show this number rising to 629 million diabetics globally [1]. In type 1 diabetes, hyperglycemia is caused by autoimmune destruction of the pancreas beta cells [2]. Type 2 diabetes mellitus (T2DM) is the more common type of diabetes where peripheral insulin resistance and compensatory increased insulin secretion may accelerate the decrease in pancreatic islet secretory function, eventually leading to insulin deficiency [2]. Diabetes is associated with several complications, including nephropathy, retinopathy, neuropathy, and atherosclerosis [2]. About 60% to 70% of all people with diabetes will eventually develop peripheral neuropathy [3]. Increasing epidemiological evidence suggests that diabetes neuropathy and T2DM may be related to increased risk of Alzheimer's disease (AD) [4]. AD is a progressive brain disorder that gradually impairs a person's memory and ability to learn, communicate, and perform daily activities [5]. An estimated 5.7 million Americans are living with AD in 2018 [6]. Considering the high prevalence and tremendous social and economic burden, it is imperative to identify an effective, safe, and inexpensive approach to delay the progression or prevent the symptoms of these diseases. However, existing antidiabetic drugs have various adverse effects, and currently, no treatment has been identified to prevent or reverse AD progression [7, 8]. Considering the biochemical association between AD and T2DM [9, 10], it is possible that there may be a common therapeutic target for AD and T2DM. Natural bioactive compounds may be alternative treatment for diabetes and a novel promising therapy for AD due to their efficacy, fewer side effects, and easy availability [11]. Researches have shown that the beneficial effects of bioactive compounds may be due to various properties such as antioxidant, anti-inflammatory, and antiapoptotic effects [11, 12]. Herein, we review the multiple beneficial effects of bioactive compounds and their underlying mechanism of actions in cell culture and animal models of AD and T2DM.

## 2. Pathophysiology of T2DM and AD

The pathophysiology of T2DM is characterized by peripheral insulin resistance, increased hepatic glucose production, and impaired *β*-cell function, eventually resulting in *β*-cell failure [13]. Insulin resistance is a condition in which cells fail to respond to normal levels of insulin that occurs mainly within the liver, muscle, and fat tissues [14]. Normally, insulin can inhibit hepatic glucose production in both postprandial and fasting states, whereas postprandial glucose production is increased in the situation of hepatic insulin resistance [15]. Elevated lipid breakdown within fat may also contribute to increased hepatic glucose production [16]. Insulin resistance initially stimulates compensatory *β*-cell proliferation and improved insulin secretion; however, long-term exposure to hyperglycemia-induced oxidative stress, endoplasmic reticulum (ER) stress, and various cytokines may contribute to *β*-cell failure due to apoptosis, autophagy, and impaired proliferation [17, 18]. The progressive degeneration of *β*-cell function leads to reduced insulin secretion and disruption of glucose homeostasis [18].

The pathological features of AD include extracellular deposition of misfolded amyloid plaques (A*β* peptide) in senile plaques, intracellular neurofibrillary tangles (NFTs), inflammation, and brain atrophy [19]. A*β*, a 38-43 amino acid residue peptide, originates from proteolysis of the amyloid precursor protein (APP) [20]. In the nondisease state, APP produces nonamyloidogenic A*β* products by *α*-secretase, but in the AD brain, A*β* is produced from APP by the sequential enzymatic actions of *β*-site APP cleaving enzymes 1 (BACE-1, a *β*-secretase) and *γ*-secretase [20, 21]. The imbalance between the production and clearance of A*β* leads to A*β* accumulation and its subsequent aggregation and neurotoxicity [22]. A*β* spontaneously aggregates into different forms, including 3-50 A*β* monomers, oligomers, fibrils, and plaques [22]. Soluble oligomers appear to be the most toxic form [23]. NFT primarily consists of hyperphosphorylated tau, which is insoluble and loses the ability to bind to microtubules, and hyperphosphorylated tau self-aggregates into toxic, helical filament structures [21].

## 3. Possible Links between T2DM and AD

T2DM and AD share many characteristics, including chronic inflammation, oxidative stress, impaired insulin signaling, insulin resistance, glucose intolerance, and cognitive impairment [9].

### 3.1. Insulin Resistance

Increasing evidence has shown that insulin deficiency and resistance, the markers of T2DM, are also important in AD pathology [24]. Moreover, it was proposed that AD may be a brain-specific form of diabetes mellitus, a “type 3 diabetes” [10]. Insulin receptors (IR) are expressed in the peripheral systems as well as central nervous system, especially in the hippocampus, which is the earliest affected structure in AD [25, 26]. The binding of insulin to IR leads to tyrosine phosphorylation and activation of insulin receptor substrate (IRS), which then activates phosphatidylinositol-3 kinase (PI3 kinase) and Akt, and Akt then mediates phosphorylation or inactivation of glycogen synthase kinase 3*β* (GSK3*β*) [20]. Impaired insulin signaling results in increased GSK3*β* activity, which causes hyperphosphorylation of tau, formation of NFTs, and increased production of A*β* [20, 27]. In the AD brain, A*β* oligomers lead to abnormal activation of tumor necrosis factor-*α* (TNF-*α*)/c-Jun N-terminal kinase pathway (JNK) and cause the inhibition of IRS1 and the disruption of insulin signaling [9, 28]. Moreover, the insulin-degrading enzyme (IDE) is responsible for the degradation of APP and A*β* [29]. Under conditions of insulin resistance, there is competition between insulin and A*β* for IDE that eventually reduces A*β* degradation [12].

### 3.2. Chronic Inflammation

Chronic inflammation may also contribute to the association between T2DM and AD. Increased levels of various proinflammatory cytokines such as TNF-*α*, interleukin-6 (IL-6), and interleukin-1*β* (IL-1*β*) have been observed in T2DM [30]. These cytokines are associated with *β*-cell damage, apoptosis, and impaired insulin secretion [31, 32]. Certain proinflammatory cytokines could also cross the blood brain barrier (BBB) and act on the central nervous system; these effects have been hypothesized to contribute to the initiation and progression of AD [33]. For example, studies have shown that increased levels of IL-1 in the brain reduced hippocampal acetylcholine (Ach) release, reduced mRNA expression of hippocampal nerve growth factor (NGF), and caused memory deficits [34]. Advanced glycation end products (AGEs) are produced via nonenzymatic glycation of amine residues on proteins, lipids, or nucleic acids by reducing sugars [35]. In diabetes, chronic hyperglycemia may promote the generation of AGEs [35], which interact with RAGE receptors, inducing the activation of different intracellular inflammatory pathways, including the nuclear factor-kappa B (NF-*κ*B) signaling cascade and inflammatory mediators such as TNF-*α*, IL-6, and C-reactive protein (CRP) [36]. AGEs may be implicated in AD pathology. In particular, A*β* has been reported to be a RAGE ligand where the binding of A*β* to RAGE promotes A*β* influx across BBB leading to the accumulation of A*β* in the brain [37, 38]. In addition, the interaction of RAGE with A*β* is associated with the activation of microglia and increased levels of oxidative stress, an augmented proinflammatory response, and neuronal injury and cell death [39].

### 3.3. Oxidative Stress

Oxidative stress, the result of the imbalance between the production of reactive nitrogen species (RNS) and reactive oxygen species (ROS), and intracellular antioxidant defense [40], is involved in the onset or progression of T2DM and AD. ROS, such as nitric oxide synthase (iNOS), cyclooxygenase-2 (COX-2), and hydroxyl radicals, are involved in causing damage to membrane polyunsaturated fatty acids, proteins, and DNA [41]. This ROS-mediated lipid peroxidation leads to loss of plasma membrane integrity and increases its permeability to Ca^2+^ [2]. Excessive ROS/RNS production plays an important role in the onset of T2DM and its complications [42]. In T2DM, increased glucose concentrations may induce glucose autoxidation, mitochondria dysfunction, and increased production of ROS [42]. The overproduction of ROS further mediates lipid peroxidation, leading to *β*-cell dysfunction, and impairs several biochemical pathways, including NF-*κ*B, JNK/stress-activated protein kinase (SARK), and p38-mitogen-activated protein kinase (p38-MAPK), which may in turn contribute to insulin resistance and late complications of T2DM [42, 43]. Oxidative stress and mitochondria dysfunction also play a critical role in AD pathogenesis [42, 44]. Neurons depend on mitochondria for ATP utilization and maintenance of calcium homeostasis; oxidative stress-induced mitochondria bioenergetic depletion can cause neuronal injury and death [44]. Moreover, mitochondria dysfunction amplifies the production of ROS, which then enhances tau hyperphosphorylation, NFT formation, and A*β* aggregation [42]. A*β* and NFT are also involved in the generation and promotion of oxidative stress [42]. All these together accelerate the progression of AD. Considering the biochemical link between T2DM and AD, it is possible that common therapeutic and preventive agents may be effective treatments for both diseases.

## 4. Effects of Bioactive Compounds on T2DM and AD and Their Mechanisms of Action

**Figure 1:**
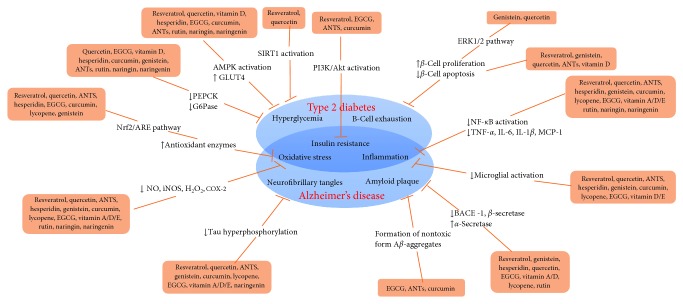
Functions of bioactive compounds in T2DM and AD pathogenesis. (1) Shared characteristics of T2DM and AD including insulin resistance, inflammation, and oxidative stress. (2) Some bioactive compounds can ameliorate hyperglycemia by activating AMPK, increasing GLUT4 translocation, inhibiting PEPCK and G6Pase activities, or activating SIRT1. (3) Some bioactive compounds can preserve functional *β*-cell mass by increasing *β*-cell proliferation or decreasing apoptosis. (4) Through activation of the PI3K/Akt pathway, some bioactive compounds improved insulin resistance. (5) Bioactive compounds attenuate oxidative stress via reducing NO, iNOS, and COX-2 levels or/and increasing the expression of antioxidant enzymes. (6) Most bioactive compounds could ameliorate inflammation which in turn improves T2DM and AD pathology. (7) Bioactive compounds can decrease A*β* production or assemble them into nontoxic aggregates, thereby decreasing formation of amyloid plaques. (8) Some bioactive compounds reduce NFT levels by inhibiting tau hyperphosphorylation. References: [46, 47, 50, 54, 55, 59, 61, 62, 64, 72, 75, 77, 81, 89, 93, 98, 101, 103, 108, 110, 116, 117, 121, 123, 126, 132, 133, 139, 140, 147, 149, 151, 152, 159, 162, 169, 175, 180, 183, 186, 189, 194–196, 200, 204, 215, 217, 227, 231, 234].

Bioactive compounds are defined as components of foods that can regulate metabolic processes in humans or animals and improve health [45]. They are found largely in vegetables, fruits, and whole grains and can be consumed daily [45]. Beneficial effects of bioactive compounds have been identified in both cell and animal studies, including decreasing inflammation, scavenging free radicals, and regulating cell signaling pathways [46, 47] (Figure 1). Because of their rich availability, safety, and few side effects, use of bioactive compounds has been proposed to reduce the incidence or delay the progression of several diseases, including T2DM and AD [11, 12]. Examples of bioactive compounds include polyphenols, carotenoids, phytosterols, prebiotics, and vitamins.

### 4.1. Polyphenols

#### 4.1.1. Resveratrol

Resveratrol is a polyphenolic compound found in grape skins, seeds, and red wines that exhibits antioxidant and anti-inflammatory properties; it also increases mitochondrial function and maintains metal homeostasis [19]. Both cell and animal studies suggested that resveratrol may have therapeutic potential in the treatment of T2DM [49]. SIRT1, an NAD^+^-dependent deacetylase, has been shown to regulate many factors that influence T2DM, and resveratrol was reported to be an activator of SIRT1 [50]. In insulin-secreting cells, resveratrol treatment potentiated glucose-stimulated insulin secretion and glucose metabolism as well as mitochondrial activity [51]. These effects were dependent on active SIRT1, which induced upregulation of key genes for *β*-cell function [51]. Moreover, resveratrol has been shown to normalize hyperglycemia, improve insulin sensitivity, and lower hepatic glucose production through the activation of SIRT1 [50]. A recent study suggested that resveratrol improved T2DM by regulating mitochondrial biogenesis, lipid metabolism, and *β* cells through activation of SIRT1 [52]. Manganese superoxide dismutase (Mn-SOD) is an important antioxidant enzyme in mitochondria, and Mn-SOD dysfunction could increase ROS production and induce tissue damage [53]. A recent study showed that resveratrol treatment ameliorated the functional and histological abnormalities and mitochondria biogenesis in the kidney of obese leptin receptor-deficient mice (db/db) mice, which is a well-accepted mouse model of type 2 diabetes, and these effects should primarily contribute to the improvement of oxidative stress via normalization of Mn-SOD function and glucose-lipid metabolism by resveratrol [53]. In addition, Lee et al. [54] reported that resveratrol treatment improved glucose tolerance, reduced high glucose-induced oxidative stress, and also attenuated *β*-cell loss in db/db mice. Further, resveratrol has been shown to reduce hyperglycemia and ameliorate dysregulated insulin signaling. Specifically, treatment of streptozotocin- (STZ-) induced diabetic rats with resveratrol increased glucose uptake through enhanced GLUT4 translocation by regulating the AMP-activated protein kinase (AMPK)/Akt/iNOS signaling pathway [55].

The beneficial effect of resveratrol in AD were also reported in both cell and animal studies. Feng et al. [56] reported that resveratrol protected P12 cells against A*β*-induced cell apoptosis through the upregulation of SIRT1 and the downregulation of rho-associated kinase 1 (ROCK1) by SIRT1 (Table 1). In addition, treatment with resveratrol in Tg2576 neuron cultures reduced the accumulation of A*β* peptides and promoted *α*-secretase activity, thereby inducing nonamyloidogenic APP processing, and these effects were partly dependent upon the activation of SIRT1 by resveratrol [57]. Activation of microglia in the brain triggers neuronal inflammation and cell death, and A*β* could trigger microglial activation by interacting with toll-like receptors (TLR) such as TLR4. It was reported that resveratrol prevented lipopolysaccharide- (LPS-, a TLR4 ligand) induced activation of murine RAW 264.7 macrophages and microglial BV-2 cells by inhibiting the TLR4/NF-K*β*/STAT (signal transducer and activation of transcription) signaling cascade [58]; therefore, the anti-inflammatory effects of resveratrol protect microglia against A*β*-induced inflammation. Moreover, the antioxidant effects of resveratrol protected rats against A*β*-induced neurotoxicity by attenuating iNOS and lipid peroxidation and increasing the production of heme oxygenase-1 (HO-1) [46]. In addition, a recent study [59] suggested that resveratrol treatment reduced microtubule-associated ubiquitin ligase (MID1) protein expression *in vitro* and *in vivo*, which in turn resulted in increased activity of microtubule-associated protein phosphatase 2A (PP2A) and further improved dephosphorylation of tau.

#### 4.1.2. Quercetin

Quercetin is a flavonoid that is naturally found in a variety of foods, including red onions, broccoli, tea, and apples [60]. Exhibiting antioxidant, anti-inflammatory, and antiapoptotic effects, quercetin has been reported to have the potential for treatment of diabetes and its complications [61–63]. Quercetin could influence glucose homeostasis in both the liver and skeletal muscle; specifically, in cultured skeletal muscle cells, quercetin increased glucose uptake through stimulation of GLUT4 translocation by activating AMPK [61]. Similarly, in hepatocytes, quercetin also activated AMPK, and this was related to the suppression of glucose-6-phosphatase (G6Pase), eventually reducing hepatic glucose production (Table 2) [61]. Youl et al. [62] reported that quercetin potentiated glucose-induced insulin secretion and protected *β*-cell function and viability from H_2_O_2_-induced oxidative damage in INS-1 cells. These effects were mediated by phosphorylation of extracellular signal-regulated kinase (ERK1/2), suggesting that ERK1/2 activation was involved in the action of quercetin [62]. Moreover, a recent study showed that quercetin treatment improved glucose and lipid metabolism and also alleviated hepatic histomorphological injury in STZ-induced diabetic rats, which probably associated with the upregulation of SIRT1 activity by quercetin and its influence on the Akt signaling pathway [63]. The vascular complications are responsible for most of the morbidity and mortality in patients with diabetes [64]. In STZ-induced diabetic rats, quercetin administration ameliorated the progression of diabetes-induced hypertension and abrogated diabetes-induced vasoconstriction [47]. These effects may be due to the inhibitory effects of quercetin on inflammatory pathways, via inhibition of NF-*κ*B and amelioration of the serum TNF-*α* and C-reactive protein (CRP) levels in the aorta of diabetic rats (Table 3) [47].

Several *in vivo* and *in vitro* studies have shown that quercetin exerts neuroprotective effects in diabetic neuropathy [65–67]. Qu et al. [65] reported that high concentrations of glucose impaired the proliferation of rat RSC96 cells and primary rat Schwan cells; inhibited the expression of beclin-1 and light chain (LC3), which are the biomarkers for autophagy; and decreased the numbers of autophagosomes in both cell types. All these effects were rescued after treatment with quercetin. Schwann cells are important for neuronal function and structure [65]; therefore, quercetin may have neuroprotective effects in diabetic peripheral neuropathy. Xia et al. [66] reported that quercetin supplement could reverse cognitive decline in mice fed a high-fat diet, possibly by altering Nrf2 signaling and eventually improving cognitive function. Additionally, a recent study indicated that quercetin reduced oxidative stress and alleviated inflammation and protein glycation in the brain of diabetic rats [67]. These effects may be related to the upregulation of glyoxalase, which is a ubiquitous cellular enzyme that participates in the detoxification of the cytotoxic byproduct of glycolysis and has been implicated in the pathogenesis of diabetic encephalopathy [67].

The beneficial effects of quercetin in AD were also confirmed in both cell and animal studies [68–70]. In cultured neurons, pretreatment with quercetin ameliorated A*β*1-42-induced protein oxidation, lipid peroxidation, cytotoxicity, and apoptosis; however, high doses were nonneuroprotective and toxic (Table 1) [68]. In Drosophila models, Kong et al. [69] found that quercetin could extend lifespan and rescue the climbing ability of AD flies, and mechanistic studies showed that cell cycle-related proteins were interrupted by A*β* accumulation and that quercetin could rescue these cell cycle-related signaling pathways. In a triple transgenic AD (3xTg-AD) mouse model, 3-month treatment with quercetin decreased extracellular *β*-amyloidosis and ameliorated microglial and astroglial activation in the brain, as evidenced by decreased levels of A*β*1-40, A*β*1-42, and BACE1-mediated cleavage of APP. Additionally, performance on learning and memory tasks was also improved (Table 4) [70]. Moreover, administration of quercetin to APPsw/PS1dE9 mice alleviated learning and memory deficits as well as decreased plaque burden compared to control mice; the protective effects of quercetin might function by reducing mitochondrial dysfunction through the activation of AMPK [71]. A recent work also suggested an anti-inflammatory role of quercetin in AD mice [72]. Specifically, quercetin treatment reduced *β*-amyloid plaque aggregation as well as decreased IL-1*β*/COX-2/iNOS proinflammatory signaling in the hippocampal CA1 region of 3xTg-AD mice [72].

#### 4.1.3. Genistein

Genistein is an isoflavone found in a variety of plants, including chickpeas, fava beans, and soybeans [73]. Several health benefits are attributed to isoflavones, and recent evidence suggests that genistein may be a potential preventative and therapeutic treatment for diabetes and AD [74–76].

Loss of functional *β*-cell mass, which decreases insulin secretion, is crucial for the development of T2DM. The mass of *β* cells is controlled by the balance between neogenesis, transdifferentiation, proliferation, and apoptosis [74]. Fu et al. [75] reported that genistein incubation induced increase of both INS-1 and human islet *β*-cell proliferation via the activation of the cAMP/PKA-dependent ERK1/2 signaling pathway (Table 2). Animal studies also showed an antidiabetic effect of genistein. Specifically, Fu et al. [75] found that induction of diabetes by STZ decreased *β*-cell mass and disrupted the cell architecture (Table 3). However, dietary supplementation of genistein improved *β*-cell mass by increasing *β*-cell proliferation and reducing apoptosis; accordingly, supplementation with genistein alleviated STZ-induced hyperglycemia and improved glucose tolerance and insulin levels [75]. Ae Park et al. [76] evaluated the antidiabetic effects of genistein on C57BL/KsJ-db/db mice, which share metabolic features that are like human T2DM. Blood glucose and HbA1c were significantly lower in the genistein groups, while glucose tolerance and the insulin/glucagon ratio were also improved in the genistein group compared to the control group [76]. In addition, the genistein supplements improved the plasma triglyceride, HDL-cholesterol, free fatty acid, and total cholesterol levels in these mice. These effects might be associated with increased hepatic glucokinase activity as well as decreased hepatic fatty acid synthase, *β*-oxidation, and G6Pase activities [76]. Therefore, genistein may exert an antidiabetic role in T2DM by improving the lipid and glucose metabolism. Furthermore, Dkhar et al. [77] reported that genistein reduced fasting glucose, inhibited cytosolic phosphoenolpyruvate carboxykinase (PEPCK), and activated AMPK and ERK1/2 pathway in alloxan-induced diabetic mice, which may in turn improve dysfunction in hepatic gluconeogenesis in T2DM. Furthermore, recent studies have shown that genistein might also be a prospective therapeutic approach for the management of T2DM complications [78, 79]. For example, Rajput et al. [78] reported that genistein treatment recovered cognitive decline in diabetic mice by modulating acetylcholinesterase, antioxidant levels, and neuroinflammation. Another interesting study indicated that genistein pretreatment improved obsessive-compulsive disorder in STZ-induced diabetic mice by increasing serotonergic neurotransmission [79].

The antioxidant, anti-inflammatory, and antiapoptosis qualities of genistein might also apply to AD. Zhou et al. [80] reported that A*β*25-35 induced inflammatory damage in BV-2 microglia, possibly through the TLR4- and NF-*κ*B-mediated signal pathway, which could be attenuated by genistein injection (Table 1). Another study indicated that pretreatment with genistein prevented the increase of inflammatory and oxidant mediators such as COX-2, iNOS, IL-1*β*, and TNF-*α* stimulated by A*β* in cultured astrocytes and that these effects may be mediated by increasing expression of peroxisome proliferator-activated receptors (PPARs) [81]. The activation of PPARs has been shown to suppress inflammation in AD [82]. Furthermore, genistein protected PC12 cells from A*β*25-35-induced neurotoxicity and neuron death by interfering with the activation of JNK, which could stimulate the transcription of the death inducer Fas ligand [83]. Moreover, recent studies indicated that genistein protected P12 cells against A*β*25-35-induced injury as well as protected AD rats against hippocampal neuron injury by regulating calcium/calmodulin-dependent protein kinase IV (CaM-CaMKIV) and tau protein expression [84, 85]. In addition, genistein as a phytoestrogen can bind estrogen receptors and impact estrogen-mediated processes [86]. In A*β*1-40-injected rats, pretreatment of genistein improved learning and memory function of rats via an estrogenic pathway and by reducing oxidative stress (Table 4) [86]. However, some studies indicated that genistein exerted toxic effects in AD pathology. For instance, in SHSY5Y cells, genistein enhanced A*β*42 accumulation by increasing mRNA expression and activities of both APP and *β*-secretase and by decreasing levels of the A*β*42-degrading enzyme IDE [87]. Considering the mixed results of the effects of genistein from *in vitro* studies, it is imperative to verify these toxic effects in experimental models.

#### 4.1.4. Epigallocatechin-3-Gallate

Epigallocatechin-3-gallate (EGCG) is a polyphenolic compound derived from a variety of plants, particularly green tea. In recent years, the beneficial effects of green tea have been studied and the health benefits are attributed to its most abundant component, EGCG [88]. EGCG exhibits strong antioxidant activity. Cytokines produced by immune cells may induce *β*-cell damage in insulin-dependent diabetes mellitus, and it is associated with the generation of iNOS and NO within the cell [89]. Han [90] reported that EGCG protected RINn5F cells against cytokine-induced *β*-cell destruction and that the molecular mechanism may involve the suppression of iNOS expression through the inhibition of NF-*κ*B activation (Table 2). Thus, EGCG may lead to enhanced pancreatic function. However, the supposed antioxidant effects of EGCG are controversial, and there is evidence suggesting that EGCG has prooxidant effects [91, 92]. For instance, Suh et al. [92] reported that EGCG mediated the production of H_2_O_2_ and triggered Fe^2+^-dependent formation of toxic radicals, which further decreased cell viability and induced apoptotic cell death in HIT-T15 pancreatic *β* cells.

Animal studies also suggest that EGCG may play a role in preventing the development of diabetes and its complications, although the evidence is not consistent [93–95]. In a db/db mouse model, EGCG consumption improved glucose-stimulated insulin secretion, oral glucose tolerance, and blood glucose in a dose-dependent manner. The increase in insulin secretion could be caused by a protective effect of EGCG on the pancreas [93]. Furthermore, the study implied that EGCG supplementation influenced the expression of genes that are involved in glucose and lipid metabolism in the liver, for example, by increasing mRNA expression of glucose kinase and decreasing mRNA expression of PEPCK, G6Pase, and fatty acid synthase (Table 3) [93]. Oršolić et al. [94] reported that administration of EGCG resulted in increased survival, decreased lipid peroxidation, and reduced DNA damage in diabetic mice and that the beneficial effects of EGCG might be associated with its antioxidant and anti-inflammatory potential. By contrast, Yun et al. [95] reported that EGCG acted as a prooxidant in *β* cells, which impaired *β*-cell function and insulin secretion by increasing oxidative stress. In biological systems, the anti- or prooxidant activity of EGCG may be different depending upon its concentration, the cellular environment, the presence of red blood cells or metal ions, and the characteristics of the cell line under investigation [92, 96, 97]; thus, additional studies are needed to determine the adverse effects of EGCG in different cell lines and pathophysiological conditions. In addition to its antioxidant property, new studies have investigated the other possible mechanism of EGCG in the treatment of T2DM [98, 99]. For instance, Zhang et al. [98] reported that EGCG improved insulin resistance in HepG2 cells through ameliorating glucose-induced inflammation and lipotoxicity via the GLUT2/peroxisome proliferator-activated receptor *γ* coactivator (PGC-1*β*)/sterol regulatory element-binding-1c (SREBP-1c)/FAS pathway.

EGCG may have the potential to improve cognitive function and attenuate the hallmarks of AD. For instance, in an *in vitro* study where cultured hippocampal neuronal cells were treated with EGCG, a protective effect against A*β*-induced neuron injury and death through scavenging ROS was found, as evidenced by decreased levels of malonyldialdehyde (MDA) and caspase, which were likely a result of decreased ROS [100]. EGCG could also prevent the development of AD by inhibiting the formation of the biomarkers of AD pathology [101]. The assembly of amyloid fibrils is involved in converting native, unfolded polypeptides A*β* into a *β*-sheet formation [102]. The presence of EGCG could directly bind the unfolded polypeptides A*β* and then assemble them into unstructured, nontoxic A*β*-oligomers instead of *β*-sheet-rich aggregates, inhibiting the fibrillogenesis of A*β* [101]. Moreover, Bieschke et al. [103] reported that EGCG could remodel the large, mature *β* fibrils into smaller, nontoxic amorphous protein aggregates, further reducing cellular toxicity (Table 1). In AD transgenic mice, chronic EGCG injections decreased A*β* levels and plaques and promoted nonamyloidogenic APP processing by increasing *α*-secretase activity (Table 4) [104]. Moreover, EGCG administered orally in drinking water (50 mg/kg, 6 months) reduced A*β* deposition, regulated the tau profile, and suppressed the phosphorylated tau isoforms in AD transgenic mice [105]. Radial-arm water-maze tests also indicated EGCG provided cognitive benefits [105]. A recent study suggested that EGCG facilitated the degradation of extracellular A*β* in astrocytes, by increasing neprilysin secretion via ERK and the phosphoinositide 3-kinase (PI3K) pathway [106]. Furthermore, Du et al. [107] indicated that EGCG attenuated the neurotoxicity in both SH-SY5Y cells and the APP/PS1 transgenic mice model, via a novel mechanism that involves suppression of ER-stress-mediated neuronal apoptosis.

#### 4.1.5. Hesperidin

Hesperidin is a flavonoid glycoside abundant in citrus fruits such as lemons and oranges. Recently, evidence from *in vitro* and *in vivo* studies has shown that hesperidin possesses beneficial effects for the prevention and treatment of T2DM and its complications, through its antioxidant, anti-inflammatory, and antidepressant properties [108–111]. In rat pancreatic islet cells, hesperidin was protective against oxidative stress induced by IL-1*β*, thereby improving the function of islet cells and restoring biosynthesis and secretion of insulin [108]. Treatment of high fat diet (HFD)/STZ-induced diabetic rats with hesperidin reduced hyperglycemia by increasing peripheral glucose uptake, which might be associated with the upregulation of GLUT4 mRNA expression (Table 2) [108]. Oral administration of hesperidin significantly decreased glucose and HbA1c levels and increased serum insulin, vitamin C, and vitamin E levels in HFD/STZ-induced diabetic rats [109]. These effects were possibly due to a decline in production of oxidants and proinflammatory cytokines such as TNF-*α* and IL-6 (Table 3) [109]. Moreover, in STZ-induced diabetic rats, hesperidin treatment attenuated retina and plasma abnormalities, including reduced retina thickness and increased blood-retina breakdown, via its antioxidant and anti-inflammatory properties, and the inhibition of the production of AGEs and elevated aldose reductase [110]. Hesperidin could attenuate experimental diabetic neuropathy. Treatment of STZ-induced diabetic rats with hesperidin significantly attenuated neuropathic pain and improved nerve conduction velocity by downregulating the production of free radical generation and proinflammatory cytokine [111]. The antidepressant effect of hesperidin was demonstrated in STZ-induced diabetic rats, which also was mediated by its antioxidant and anti-inflammatory activities as well as increased neurogenesis [112]. Furthermore, a recent study implied the protective effects of hesperidin in diabetic nephropathy, possibly through the inhibition of transforming growth factor-*β*1- (TGF-*β*1-) integrin-linked kinase- (ILK-) Akt signaling [113].

Additionally, several studies provided evidence that hesperidin may be a novel therapeutic agent for the treatment of AD [114–116]. In PC12 cells, hesperidin protected cells against A*β*25-35-induced cytotoxicity and apoptosis by attenuating mitochondria dysfunction [114]. Further study indicated that hesperidin mediated the voltage-dependent anion channel 1- (VDAV-1-) regulated mitochondria apoptotic pathway [114]. Huang et al. [115] reported that hesperidin administration ameliorated A*β*1-42-impaired glucose utilization, partly by decreasing A*β*-induced cellular autophagy in neuro-2A cells (Table 1). In APP/PS1 mice, intragastric administration of hesperidin improved learning and memory deficits by attenuating inflammation and oxidative stress through inhibition of RAGE/NF-*κ*B signaling and activation of Akt/Nrf2 signaling (Table 4) [116]. Moreover, in the transgenic APP/PS1-21 mice, hesperidin treatment significantly restored deficits in nesting and social interactions and attenuated A*β* deposition, microglial activation, and TNF-*α*, iNOS, and IL-1*β* levels in the brains of mice [117]. These results suggested that reduced A*β* deposition and alleviation of neuroinflammatory reactions by hesperidin might contribute to the improvement of behavior [117]. Taken together, studies suggest that hesperidin might be a potential candidate for the treatment and prevention of T2DM and AD; however, more studies on the clinical effects of hesperidin should be performed.

#### 4.1.6. Anthocyanins

Anthocyanins (ANTs) are flavonoids responsible for the blue, red, and purple colors of vegetables, fruits, and flowers [118]. Most ANTs act as strong antioxidants, which may contribute to their antidiabetic properties. Zhang et al. [119] reported that ANTs from Chinese bayberry extract upregulated HO-1 expression via activation of PI3K/Akt and ERK1/2 signaling in INS-1 cells. As a result, ANTs protect cells against H_2_O_2_-induced *β*-cell injury. Furthermore, Zhang et al. [120] found that pretreatment with ANTs attenuated H_2_O_2_-mediated *β*-cell autophagy by activating the antioxidant transcription factor Nrf2. Additionally, in HepG2 cells, mulberry ANT extract was reported to mitigate insulin resistance via activation of PI3K/Akt pathways (Table 2) [121]. In STZ-induced diabetic rats, injection of the ANT pelargonidin improved serum insulin levels, normalized elevated blood glucose levels, and glucose tolerance. It also relieved oxidative stress, including the hemoglobin- (Hb-) induced iron-mediated oxidative reaction, by releasing iron from the Hb and decreasing Hb glycation (Table 3) [122]. ANTs from black soybean seed coats also yield antidiabetic effects such as decreasing blood glucose levels and improving hemodynamic parameters and insulin levels in STZ-induced diabetic mice [123]. These effects were partly due to the regulation of GULT4 transporter, the activation of the phosphorylation of insulin receptor, and the prevention of pancreatic apoptosis [123]. Recently, Luna-Vital et al. [124] demonstrated that ANT from purple corn improved insulin secretion and hepatic glucose uptake *in vitro*, by enhancing the activity of the free fatty acid receptor-1 (FFAR1) and glucokinase.

Growing evidence suggests that ANTs may have beneficial effects on AD. Shih et al. [125] reported that exposure of A*β*1-40 and A*β*25-35 to neuro-2A cells resulted in ROS formation, the perturbation of calcium balance, and influenced the expression of genes involved in apolipoprotein E (ApoE) metabolism. All these effects could be blocked by ANT treatment, eventually leading to reduction of A*β*-induced neurotoxicity (Table 1). In addition, treatment of neuro-2A cells with *Vaccinium myrtillus* anthocyanoside, a heterogenous mixture of ANTs, promoted the formation of nontoxic forms of A*β* aggregates instead of the toxic amyloid fibrils [126]. The molecular mechanism may involve the direct binding between ANT and A*β* molecules to suppress amyloid fibril formation, a function similar to that of EGCG [101]. Moreover, APdE9 mouse fed a diet rich in ANT from bilberry or blackcurrant supplementation showed altered APP processing and A*β* levels. Specifically, both bilberry and blackcurrant extracts decreased APP-C-terminal fragment levels in the cerebral cortex compared to animals fed the control diet [127]. Soluble A*β*40 and A*β*42 levels were decreased in bilberry-fed mice but not blackcurrant-fed mice, and by contrast, the ratio of insoluble A*β*42/40 was significantly decreased in blackcurrant-fed mice but not in bilberry-fed mice. Both berry diets attenuated behavioral abnormalities of aged mice as compared to control diet-fed mice (Table 4) [127].

Although several studies have demonstrated the beneficial effects of ANTs on T2DM and AD, further studies are needed to clarify what type of ANT is most appropriate for a given purpose, because different sources of ANTs were used in studies.

#### 4.1.7. Curcumin

Curcumin is a polyphenolic compound extracted from the dried roots of turmeric plants [128]. More than 500 published articles were retrieved when searching the PubMed database using the terms “curcumin and diabetes.” In these articles, various pharmacological properties of curcumin were noted. Its antioxidant and anti-inflammatory properties are the most well known [129]. Hepatic stellate cells (HSCs) are the major effectors during T2D-associated hepatic fibrogenesis [130], and AGEs have been shown to induce gene expression of RAGE in HSCs, which could stimulate the activation of HSCs [130]. Lin et al. [129] reported that curcumin eliminated the stimulation of AGE probably by increasing gene expression of PPAR*γ*, which attenuated the gene expression of RAGE, and alleviated the oxidative stress. Furthermore, curcumin protected pancreatic islets against STZ-induced oxidative stress by scavenging free radicals [131]. Curcumin increased islet viability and insulin secretion and decreased ROS concentration and the generation of NO as well as prevented the overactivation of poly ADP-ribose polymerase-1 (Table 2) [131]. In db/db mice, oral curcumin mitigated hyperglycemia-induced liver and kidney damage through normalization of mitochondrial function, by suppressing NO synthesis and lipid peroxidation [132]. Another study indicated that oral treatment with curcumin decreased body weight and blood glucose levels and increased plasma insulin levels [133]. In addition, curcumin attenuated hyperglycemia-induced oxidative stress, ER stress and its related inflammation, and protected *β* cells from apoptotic damage. These effects might be associated with the activation of HO-1 and the inhibition of NF-*κ*B signaling through a PI3K/Akt-mediated pathway, as well as the suppression of multiple apoptotic signaling (ER-mediated and mitochondrial-dependent or mitochondrial-independent apoptotic pathways) (Table 3) [133]. Curcumin has been shown to exhibit antihyperlipidemic activity. Pari and Murugan [134] reported that treatment of STZ-induced diabetic rats with intragastric tetrahydrocurcumin, one of the active metabolites of curcumin, resulted in a significant reduction of serum-free fatty acids, triglycerides, VLDL, LDL, and cholesterol and an increase of HDL cholesterol. Furthermore, curcumin inhibited hepatic gluconeogenesis by inhibiting hepatic G6Pase and PEPCK activities and activating AMP kinase [135]. Moreover, a recent study showed that curcumin improved insulin resistance and also ameliorated the metabolic disorder of glucose and lipid in T2DM rats; these effects might be associated with the reduction of the free fatty acid and TNF-*α* in serum [136].

Curcumin also emerged as a promising therapeutic option for AD. Huang et al. [137] reported that curcumin inhibited A*β*-induced tau hyperphosphorylation in human neuroblastoma SH-SYSY cells, which is involved in the phosphatase and tension homolog (PTEN)/Akt/GSK3*β* pathway. Qian et al. [138] showed that curcumin treatment protected P12 cells from A*β*-induced reduction in MDA production, cell viability, and apoptosis, by increasing the expression of the N-methyl-D-aspartate receptor (NMDAR) subunit NR2A. In an Alzheimer transgenic APPsw mouse model, curcumin decreased overall insoluble and soluble amyloid and plaque burden, and it reduced oxidative stress and suppressed the inflammatory cytokine IL-1*β* and astrocytic inflammatory marker glial fibrillary acidic protein (GFAP) (Table 4) [139]. Moreover, increasing evidence suggests that curcumin could bind A*β* and shift its aggregation pathway. For instance, Rao et al. [140] found that curcumin binding to A*β* promoted the formation of nontoxic forms of A*β* aggregates. Similarly, another study indicated that curcumin could bind to highly aggregated A*β* as well as to abnormal tau protein in the brain of aged AD animals; therefore, curcumin might be used as a specific marker for A*β* detection [141]. Overall, these finding highlight the potential utility of curcumin in T2DM and AD protection fields. However, there are some limitations to its therapeutic use, including poor bioavailability, rapid metabolism, and rapid systemic elimination [142]. Additional approaches are needed to enhance its bioavailability, and more clinical trials are needed to confirm its potential in prevention of AD and T2DM.

#### 4.1.8. Rutin

Rutin is a flavonoid in many vegetables and fruits, such as apples, figs, buckwheat, and asparagus [143]. It has a wide range of biological effects including antioxidant, anti-inflammatory, antihyperglycemic, and neuroprotective [144, 145]. All these properties support the potential of rutin to prevent or treat diabetes and its complications. For example, in nicotinamide- (NA-) STZ-induced diabetic rats, administration of rutin significantly ameliorated glucose tolerance; decreased serum glucose levels; produced improvement of the increased serum lipid variables, such as LDL-cholesterol, VLDL-cholesterol, triglycerides, and serum total lipids; and also improved the oxidative stress [146]. The possible mechanisms for the antihyperglycemic and antihyperlipidemia effect of rutin were investigated in further studies. It has been shown that rutin decreased the activity of G6Pase and glycogen phosphorylase, as well as increased the activity of hepatic hexokinase activity; therefore, rutin may reduce hepatic glucose output [146]. Furthermore, the decrease in glucose level can be achieved by improving glucose uptake by tissues [145]. Hsu et al. [147] reported that rutin reduced blood glucose level in insulin-resistant mice through enhancement of insulin-dependent receptor kinase (IRK) activity and GLUT4 translocation (Table 3). In adipose tissue and skeletal muscle, rutin has been shown to increase expression of PPAR*γ*, which further improve insulin resistance, affect insulin sensitivity, and improve glucose uptake [146, 148]. Moreover, rutin treatment increased *β*-cell viability and reduced the glucotoxicity through activating AMPK and IRS2 signaling [149]. Furthermore, it has been shown that rutin improved insulin secretion in isolated rat pancreatic islets [146]. Taken together, the antihyperglycemic effect of rutin may be achieved by increasing glucose uptake by peripheral tissue, improving insulin resistance, suppressing gluconeogenesis in the liver, and stimulating insulin secretion.

In addition to antihyperglycemia and antihyperlipidemia, rutin also exhibits antidiabetic effects by decreasing oxidative stress and suppressing the inflammatory cytokine in STZ-induced diabetic rats [150]. Moreover, a very recent study showed that rutin exhibited protective effect on the liver of db/db mice by activating the IRS2/PI3K/Akt/GSK3*β* signal pathway, improving hepatocyte proliferation, and decreasing generation of AGEs [151]. Overall, several cell and animal studies support the beneficial effects of rutin on T2DM. Further clinical studies are suggested to evaluate the efficiency and safety of rutin.

The therapeutic potential of rutin for AD has also been shown in both cell and animal studies [152, 153]. The possible mechanisms involved are eliminating the inflammatory component of neurodegeneration, decreasing oxidative stress which relates to neuronal cell loss, and preventing A*β* aggregation [154]. For example, in APPswe (APP Swedish mutation) cells, rutin treatment prevented A*β*25-35 fibril formation and inhibited BACE activity [152] (Table 1). Furthermore, rutin ameliorated the neurotoxic effect, including declined cell viability and reduced GSH levels induced by overexpression of APP in APPswe cells [152]. Similarly, Wang et al. [153] indicated that rutin inhibited A*β*42 fibrillization and improved A*β*42-induced cytotoxicity in SH-SY5Y cells. Additionally, rutin attenuated mitochondrial damage and decreased the generation of ROS, GSSG, NO, iNOS, and proinflammatory cytokines, as well as enhanced the activities of SOD and catalase [153]. Moreover, a recent study showed that *Nelumbo nucifera* extracts exhibited protective effect on A*β*-induced apoptosis in PC12 cells; further purification of these extracts identified them to be flavonoids, such as rutin [155]. In A*β*-injected rats, administration of rutin significantly enhanced memory retrieval compared to the control group, possibly through activation of the MAPK pathway and brain derived neurotraphic factor (BDNF) gene expression and reduction of oxidative stress and neurotoxicity induced by A*β* (Table 4) [156]. Furthermore, Choi et al. [157] found that the impaired cognition and memory of A*β*-induced AD mouse was alleviated by oral administration of rutin.

#### 4.1.9. Naringin

Naringin, a flavonoid mostly found in grape fruit and related citrus species, has been reported for its antioxidant, anti-inflammatory, and antihyperglycemic properties [158, 159]. Recently, several new investigations indicated that naringin could improve T2DM and mitigate the severity of T2DM complications [159–161], and the underlying mechanism has been elucidated. In NA/STZ-induced type 2 diabetic rats, naringin produced a significant amelioration of the serum glucose level and lipid profile, such as LDL-cholesterol, LDL, and free fatty acids (Table 3) [159]. These effects may be mediated by elevating liver G6Pase and glycogen phosphorylase activities, improving the insulin secretory response, and enhancing the expression of GLUT4, insulin receptor, and adiponectin as well as decreasing oxidative stress [159]. In *in vitro* studies, it has also been shown that naringin protected the cell against high glucose-induced damage. For instance, Chen et al. [160] reported that naringin inhibited the high glucose-induced inflammatory reaction by mediating the nucleotide-binding and oligomerization domain-like receptor family pyrin domain-containing 3 (NLRP3) inflammasome in the rat mesangial cell. Furthermore, Li et al. [161] indicated that naringin protected the human endothelial cell against high glucose-induced damage through inhibition of oxidation, downregulation of the chemokine (C-X3-C motif) ligand 1 (CX3CL1), and improvement of mitochondrial function (Table 2).

Furthermore, several studies have demonstrated the beneficial effect of naringin on diabetic complications including diabetes-associated anemia, kidney damage, cognitive decline, and atherosclerosis [162–164]. For instance, Mahmoud [162] reported that naringin protected HFD/STZ diabetic rats from diabetes-associated anemia by decreasing proinflammatory cytokine production and stimulating adiponectin expression. Sharma et al. [163] demonstrated that naringin attenuated hepatic steatosis and kidney damage, and also ameliorated insulin resistance and *β*-cell dysfunction by decreasing oxidative stress and inflammation through upregulation of PPAR*γ*, heat shock protein-27, and heat shock protein-72. In addition, the effects of naringin on oxidative stress, proinflammatory factors, and the PPAR*γ* signaling pathway may be involved in ameliorating cognitive deficits in the type 2 diabetic rat model [164]. Recently, an interesting study showed that naringin exhibited antiatherogenic effect in a T2DM rat model; the underlying mechanism may be involved in the enhancement of HDL-mediated reverse cholesterol transport and the improvement of paraoxonase activity [165].

The potent neuroprotective effects of naringin have been well characterized, and increasing attention has been focused on its protective effects on AD. In an APP/PS transgenic mouse model, naringin consumption enhanced learning and memory ability of mice, ameliorated cognitive deficits, and also reduced senile plaque formation and reversed glucose uptake defect in the brain. The inhibition of GSK3*β* activity may be the possible mechanism [166]. Another study suggested that the enhancement of CaMKII activity may be one of the mechanisms by which naringin improved cognitive function in the AD mouse model (Table 4) [167]. Moreover, naringin treatment restored intracerebroventricular STZ-induced cognitive deficits in rats, the mitigation of mitochondrial dysfunction mediated oxidative stress, and the suppression of acetylcholinesterase activity and the TNF-*α* level by naringin may contribute to its function on cognitive impairment [168]. A recent study has investigated the effects of naringin dihydrochalcone (NDC) on neuropathology in APP/PS1 transgenic mice [169]. NDC is a naringin derivative and acts as an artificial sweetener with antioxidant activity in food and medicine [170]. The results suggested that NDC attenuated A*β* deposition and neuroinflammation and enhanced neurogenesis as well as ameliorated cognitive deficits in AD mice [169].

#### 4.1.10. Naringenin

Naringenin is a flavonoid abundantly found in citrus fruits such as oranges, lemons, grapefruits, and tomatoes [171]. In recent years, there has been increased attention on the benefits of naringenin on T2DM and its complications. In STZ-induced diabetic rats, oral administration of naringenin decreased the blood glucose level, normalized LDL, and VLDL concentrations and also normalized oxidative stress parameters in both the liver and pancreas; these effects may be attributed to the increased expression of mRNA and protein levels of GLUT4 and PPAR*γ* by naringenin [172] (Table 3). Many studies have been designed to evaluate the role of naringenin in diabetes-associated complications, such as nephropathy, cardiac hypertrophy, vascular disease, hepatotoxicity, and neuropathy [173–175]. For instance, Kapoor et al. [173] demonstrated that the altered activity of liver and kidney enzymes, altered antioxidant status, increased generation of ROS, mitochondria dysfunction, and increased expression of apoptotic proteins could induce liver damage and diabetic hepatopathy in diabetic rats; all these effects were rescued after naringenin treatment; therefore, naringenin has potential for the management of diabetic hepatopathy. Roy et al. [174] showed that naringenin alleviated renal impairment and structural changes such as glomerulosclerosis in STZ-induced diabetic rats, possibly through downregulation of TGF-*β*1 and IL-1 by reducing oxidative stress, modulating proinflammatory cytokine production and apoptotic events. Moreover, researchers found that naringenin ameliorated high glucose-induced endothelial dysfunction by decreasing oxidative stress and apoptosis via the ROS/caspase-3 and NO pathway in endothelial cells [175]. Furthermore, naringenin acted as an antioxidant and cholinesterase inhibitor and ameliorated diabetes-induced memory dysfunction in rats [176]. Moreover, in a recent study, naringenin has been shown to improve cardiac hypertrophy in diabetic mice; these effects may be related to the upregulation of cytochrome P450 2J3 and the activation of PPARs [177]. Overall, the beneficial effects of naringenin on diabetes and its complications have been investigated, partly through its antioxidant, anti-inflammatory, and antiapoptotic properties.

In recent years, a few studies have explored the possible role of naringenin in prevention and treatment of AD. For instance, in an AD rat model, the expression of A*β*40 and A*β*42 were downregulated, and the learning and memory ability were improved after naringenin administration [178]. Another study has investigated the underlying mechanisms in A*β*-injected rats; the results suggested that naringenin pretreatment alleviated A*β*-induced impairment of memory and learning through downregulation of lipid peroxidation and apoptosis and also through mediation of the estrogenic pathway (Table 4) [179]. In PC12 cells, naringenin suppressed A*β*25-35-induced nerve damage by improving cell viability, stimulating Akt and GSK3*β* activation, inhibiting cell apoptosis, and regulating the estrogen receptor [180]. The collapsin response mediator protein-2 (CRMP-2) has been implicated in the pathogenesis of AD; phosphorylation leads to its inactivity, which in turn inhibits axonal outgrowth and results in neuronal loss and memory deficits [181, 182]. A recent study reported that naringenin could bind to CRMP-2 then decrease its phosphorylation, which in turn alleviates AD-like pathology [181]. Even though naringenin has a wide range of activities, due to its low water solubility and poor bioavailability, the clinical development of naringenin has been hampered [182]. A recent study has developed naringenin-loaded nanoemulsions, which protected SH-SY5Y cells against A*β*-induced neurotoxicity, possibly by reducing amyloidogenesis and tau hyperphosphorylation; also, it showed a better neuroprotective effect than free naringenin [183]. Overall, naringenin might be a potential agent for treatment of AD; further studies are needed to identify more underlying mechanisms and develop an optimal form of naringenin.

### 4.2. Carotenoid

#### 4.2.1. Lycopene

Lycopene is a carotenoid occurring naturally in tomatoes and pink grapefruits that is responsible for the red color [184]. Although there is little evidence regarding the possible antidiabetic effects of lycopene from *in vitro* studies, many *in vivo* studies have shown the beneficial effects of lycopene on diabetes and its associated complications [185–187]. Ali and Agha [185] conducted a study with STZ-induced diabetic rats where supplementation with lycopene (Table 3) caused a dose-dependent decrease in H_2_O_2_, NO, and lipid peroxidation, as well as increased activity of antioxidant enzymes, which further contributed to the decreased glucose levels, increased insulin levels, and improved serum lipid profiles (Table 3). The antioxidant properties of lycopene also have been shown to rescue diabetic endothelial dysfunction in STZ-induced diabetic rats [186]. To study the specific therapeutic effect of lycopene on diabetic nephropathy, Li et al. [187] conducted a study with STZ-induced diabetic rats. The results indicated that lycopene protected kidneys against diabetes mellitus-induced morphological destruction and function impairments by improving oxidative status, increasing Akt phosphorylation, and regulating connective tissue growth factor. Another study indicated that lycopene ameliorated renal function by interrupting the AGE-RAGE axis [188]. In addition, lycopene has been tested for its ability to attenuate diabetes-associated cognitive decline. Kuhad et al. [189] reported a dose-dependent response to chronic treatment with lycopene that alleviated cognitive impairment and cholinergic dysfunction, decreased NO and TNF-*α*, and increased acetylcholinesterase activity in STZ-induced diabetic rats. The dysfunction of endothelial progenitor cells (EPCs) has been implicated in diabetes-associated vascular complications [190]; Zeng et al. [191] showed that lycopene ameliorated AGE-induced EPC apoptosis and oxidative autophagy, further impairing the number and function of EPCs. Therefore, lycopene may have potential to improve T2DM vascular complications. Taken together, the antidiabetic function of lycopene might be associated with its antioxidant and anti-inflammatory properties.

Recent interest has focused on lycopene as a potential useful agent in the management of AD. The antioxidant, anti-inflammatory, and antiapoptotic effects of lycopene may directly link to its neuroprotective function. In primary cultured rat cortical neurons, pretreatment with lycopene attenuated A*β*25-35-induced neurotoxicity, as evidenced by improved cell viability and decreased rate of apoptosis in a dose-dependent manner; these effects were attributed to the inhibition of the A*β*25-35-induced generation of ROS and mitochondrial membrane potential collapse (Table 1) [192]. Furthermore, Qu et al. [193] reported that lycopene protected mitochondria against A*β*-induced damage in cultured rat cortical neurons, and its effects in part resulted by decreasing mitochondrial oxidative stress and improving mitochondrial function. Chen et al. [194] found that lycopene could reduce A*β*1-42 secretion by inhibiting APP expression in APPsw cells. Moreover, administration of oral lycopene improved A*β*-induced learning and memory in an AD mouse model. Mitigation of NF-*κ*B activity and the downregulation of TNF-*α* and IL-1*β* by lycopene might be the underlying mechanism (Table 4) [195]. In tau transgenic mice expressing P301L mutation, lycopene supplementation ameliorated the memory impairment by inhibiting oxidative stress as well as attenuating tau hyperphosphorylation [196]. Although several studies have assessed the antidiabetic and neuroprotective function of lycopene in cell and animal models, few clinical studies have been performed. To establish proper dietary recommendations, large-scale human studies are necessary.

### 4.3. Vitamins

#### 4.3.1. Vitamin A

Vitamin A or retinol is an essential dietary nutrient that is necessary for vision, reproduction, and normal growth. Intracellularly, retinol can be converted to retinal all-trans-retinoic acid (RA) or 9-cis-retinoic acid [197]. The potential mechanisms through which vitamin A can impact T2DM include chelation of oxide radicals, increasing insulin sensitivity, regeneration of *β* cells, and regulation of obese and adipose biology [197]. For instance, it was suggested that all-trans-RA could improve insulin signaling by inhibiting protein kinase C (PKC) activity through binding to PKC isozymes. PKC was found to be elevated in diabetes and abrogated insulin signaling [197]. RA also increased insulin secretion and insulin mRNA levels in cultured islets, by increasing pancreatic glucokinase through activation of the glucokinase promoter (Table 2) [198]. Moreover, retinol and RA are positive regulators of uncoupling protein 1 (UCP-1), and the overexpression of UCP-1 could improve skeletal muscle glucose transport and insulin resistance [199]. Additionally, Berry and Noy [200] reported that all-trans-RA suppressed obesity and insulin resistance by inducing expression of PPAR*β*/*δ* and retinoid acid receptor (RAR) genes (Table 3). A recent study [201] suggested that vitamin A-deficient diet-fed rats displayed reduced stearoyl-CoA desaturase 1 (SCD1) and monounsaturated fatty acid levels, which in turn increase ER stress-mediated apoptosis and alter the structure and function of the pancreas. However, there is controversy about the effects of vitamin A on the treatment of T2DM. It was reported that the metabolic availability of retinoid could be reversed by insulin treatment [202]; therefore, vitamin A may not be an effective intervention for diabetic individuals with altered retinoid biology. Additionally, large-dose intakes of vitamin A interfere with bone metabolism and are associated with osteoporosis [197].

Vitamin A could also play an important role in nerve regeneration, neural development, neural plasticity, and neurodegenerative diseases, including AD [203]. Several studies have been shown the potential effects of vitamin A on amyloid pathology, neurotransmission, oxidative stress, and inflammation. *In vitro*, in a dose-dependent manner, vitamin A inhibited oligomerization and fibrillation of A*β*40 and A*β*42 (Table 1) [204]. Vitamin A was also reported to regulate the expression of genes involved in the production of A*β*, including BACE1 and presenilin 1/2 [205, 206]. Treatment of APP/PS1 transgenic mice with all-trans-RA attenuated A*β* deposit accumulation and tau hyperphosphorylation and improved spatial learning and memory when compared with the control mice (Table 4) [207]. Deficiency in cholinergic transmission is the major underlying feature of AD, which may be attributed to the decreased expression of choline acetyltransferase (ChAT). It was reported that all-trans-RA administration upregulated the expression and activity of ChAT in a neuronal cell line [208]. Zeng et al. [209] established a marginal vitamin A deficiency (MVAD) rat model from maternal MVAD rats, then injected rats with A*β*1-42; the results showed that MVAD feeding exacerbated A*β*1-42-induced learning and memory deficits; therefore, long-term MVAD may result in an increased risk of AD. In contrast, a recent study [210] showed that increased availability of retinol at levels above the cellular physiological concentrations increased oxidative stress; the levels of *α*-synuclein, A*β*, and tau phosphorylation in human SH-SY5Y neuronal cell term MVAD may result in an increased risk of AD.

#### 4.3.2. Vitamin D

Vitamin D exists in two forms, cholecalciferol (VD_3_) and ergocalciferol (VD_2_). VD_3_ can be obtained from diet or synthesized in the skin from 7-dehydrocholesterol during exposure to solar UVB radiation. In the kidney, it is converted to 1,25-(OH)_2_ VD_3_, the active form of vitamin D [211]. Vitamin D is mediated by its nuclear receptor, vitamin D receptor (VDR). Vitamin D plays a crucial role in modulating the risk of T2DM by influencing insulin sensitivity, *β*-cell function, and inflammation [146, 149]. In peripheral insulin-target cells, vitamin D may affect insulin sensitivity by stimulating the expression of insulin receptor through interaction with VDR or by activating PPAR*κ* [212, 213]. Calcium is important for insulin-mediated intracellular processes [214], and vitamin D could regulate intracellular and extracellular calcium concentrations to affect insulin sensitivity. Moreover, vitamin D may promote *β*-cell survival by modulating the generation and activity of cytokines through the downregulation of NF-*κ*B (Table 2) [215] or the Fas-related pathway [216]. A recent study suggested that vitamin D increased glucose-stimulated insulin secretion by enhancing calcium influx through upregulation of expression of R-type voltage-gated calcium channel (VGCC) gene in mouse and human islets [217]. Treatment of STZ-induced diabetic mice with a vitamin D-supplemented diet decreased the fasting blood glucose levels, increased insulin levels, and restored pancreatic islets damaged by STZ [218]. Meerza et al. [219] (Table 3) also demonstrated that the treatment of 1,25-(OH)_2_ VD_3_ significantly changed blood calcium and glucose concentrations, as well as the activities of glucose metabolic enzymes, including G6Pase, hexokinase, and fructose 1,6-bisphosphatase (FBPase) in type 2 diabetic mice.

Recent studies showed that VDR is widely expressed in the brain [220]. Prospective studies have reported that vitamin D deficiency was associated with increased risk of AD [221]. Therefore, vitamin D may exhibit neuroprotective functions such as regulation of neurotransmitters, NGF synthesis, calcium homeostasis, A*β* metabolism, oxidative stress, and inflammation [222, 223]. For instance, NGF signaling interruption has been shown to upregulate APP and *β*-secretase leading to an increased level of A*β* [224]. In mouse fibroblasts, 1,25-(OH)_2_ VD_3_ was reported to induce NGF expression by increasing AP-1 binding activity in the NGF promoter (Table 1) [222]. Furthermore, vitamin D could stimulate A*β* clearance by macrophages of AD patients [223]. In the TgCRND8 mouse model of AD, treatment of vitamin D resulted in reduced soluble and insoluble plaque-related A*β*, primarily in the hippocampus in which the VDR is abundant, and improved memory function [225]. In addition, a recent study reported that vitamin D supplementation was efficient in improving endogenous neurogenesis and working memory in transgenic AD-like male mice when administered before the onset of the symptoms, while in female mice, vitamin D was efficient when delivered during the symptomatic phase of the disease [226]. Overall, further studies are needed to test the safety and efficacy of long-term use of vitamin D and to identify what type of vitamin D supplement is more beneficial for T2DM and AD patients, according to their age, gender, and disease stage.

#### 4.3.3. Vitamin E

Vitamin E is an important component of the antioxidant system in all body tissues, and *α*-tocopherol is the most active form. Due to its antioxidant activity, vitamin E has been considered to be a promising therapeutic option for AD and T2DM. *In vivo*, STZ-induced diabetic rats were reported to have significantly decreased glucose levels and improved activities of antioxidant enzymes such as catalase, glutathione peroxidase, and glutathione reductase after supplementation with vitamin E [227]. In addition, vitamin E supplementation ameliorated alloxan-induced mouse hyperglycemia by enhancing insulin secretion from the alloxan-treated islets (Table 3) [228]. However, the results from human studies are inconsistent, and a systematic review concluded that there were no beneficial effects of vitamin E supplementation in improving glycemic control in the full set of T2D patients. It was effective only in patients with low-serum vitamin E concentrations or inadequate glycemic control at baseline [229].

Previous studies have reported that the antioxidant and anti-inflammatory properties of vitamin E contribute to its neuroprotective effects. An animal study showed that depletion of *α*-tocopherol resulted in increased lipid peroxidation, which in turn impaired A*β* clearance from the brain and blood of AD transgenic model mice, eventually causing A*β* accumulation in the brain and plasma of mice [230]. Moreover, both *in vivo* and *in vitro* studies showed that vitamin E protects against the formation of A*β*-induced tau phosphorylation through the inhibition of the activation of p38-MAPK by reducing oxidative stress (Table 1) [231]. Beyond antioxidant activity, recent studies have identified the role of vitamin E in gene regulation, signaling, and membrane fluidity [232]. Rats fed a vitamin E-deficient diet showed changes in hippocampus gene expression. These genes were associated with apoptosis, NGF, A*β* clearance, and the onset or progression of AD [233]. For example, the expression of APP binding protein 1, which binds and stabilizes APP, was decreased after treatment with a vitamin E-deficient diet [233]. In addition, *α*-tocopherol was shown to inhibit the activation of PKC and improve the activity of PP2A, an enzyme that is implicated in AD pathology [234, 235]. A recent study [236] investigated that vitamin E had positive characteristics with respect to AD in neuronal cell lines, including reduction of ROS, cholesterol, and cholesterol ester levels; however, it also had negative effects such as enhancement of A*β* production and inhibition of A*β* degradation. Overall, *in vivo* and *in vitro* studies have established plausible effects of vitamin E on AD pathology, but more clinical research are needed for conclusive results.

## 5. Conclusion

T2DM and AD are complex disorders with high prevalence and heavy social and economic burdens. The ineffectiveness of the current therapeutic agents in management of AD and long-term diabetes complications require the development of safe and effective complementary approaches. The therapeutic potential of various bioactive compounds such as resveratrol, curcumin, and lycopene has attracted the interest of researchers. It is important to identify the molecular mechanisms underlying the antidiabetic and neuroprotective effects of bioactive compounds in cell cultures and animal models of T2DM and AD. Published data indicate that there might be beneficial effects of bioactive compounds on decreasing hyperglycemia, enhancing insulin secretion, improving *β*-cell function, decreasing A*β* accumulation, and improving cognitive function in those afflicted. The mechanisms of action may involve their antioxidant, anti-inflammatory, and antiapoptotic properties. Moreover, some studies of these bioactive compounds have yielded controversial results, which may be attributed to different experimental designs, dosages, and types of bioactive compounds examined. Additional carefully designed clinical trials are needed to provide better evidence for the potential therapeutic application of bioactive compounds in the treatment of T2DM and AD.

## Figures and Tables

**Table 1:** Effects of bioactive compounds on Alzheimer's disease (*in vitro* studies).

Bioactive compounds	Models	Treatment	Effects	Specific mechanism of action	Reference
*Polyphenols*					
Resveratrol	PC12 cells	12.5, 25, 50, and 100 *μ*M, 2 h prior to the A*β*25–35, 24 or 48 h	↑Cell viability ↓A*β*25–35-induced intracellular Ca2+ level ↓Cell apoptosis	↑SIRT1 ↓ROCK1	[56]
Quercetin	Primary rat neuronal cells	Low dose: 5 and 10 *μ*M, High dose: 20 and 40 *μ*M, 24 h	↓A*β*1-42-induced apoptotic cell death and cell toxicity at low dose ↑Toxic at high dose	↓Lipid peroxidation ↓Oxidative stress	[68]
Genistein	BV-2 microglia cells	50 *μ*M, 2 h before incubation with A*β*25–35, 24 h	↑Cell viability ↓A*β*25–35-induced inflammatory damage	↓The expression of TLR4, NF-*κ*B, ↓The activity of NF-*κ*B	[80]
EGCG	HEK-293 cells	15 and 20 *μ*M, 1-3 days	Convert large, mature amyloid-*β* fibrils into smaller, amorphous, and nontoxic aggregates	Directly binds to *β*-sheet-rich aggregates and mediates the conformational change	[103]
Hesperidin	Neuro-2A cells	20 *μ*M, 6 h pretreatment before exposure to A*β*1-42	↓A*β*-induced impairment of insulin signaling and glucose uptake	↓A*β*-induced autophagy ↑IRS-PI3K-Akt signal transduction	[115]
Anthocyanins	Neuro-2A cells	50 *μ*M malvidin or ononin with A*β*, 48 h	↓A*β*-induced neurotoxicity, cell cycle arrest	↑Ca^2+^ homeostasis ↓A*β*-induced ROS	[125]
Curcumin	Macrophages from AD patients	0.1 *μ*M	↓A*β* aggregates	↑A*β* uptake by macrophages	[238]
Rutin	APPswe cells	1, 5, and 10 *μ*M	↓The formation of A*β* fibrils and disaggregated A*β* fibrils ↓Neurotoxicity	Free-radical scavenger activity	[152]
Carotenoid					
Lycopene	Rat cortical neurons	0.1, 1, 2, and 5 *μ*M, 4 h pretreatment before exposure to A*β*	↑Cell viability ↓Apoptotic rate	↓A*β*-induced ROS ↓Mitochondrial membrane potential depolarization	[192]

*Vitamins*					
Vitamin A	—	100, 150, and 250 *μ*M retinoid acid	↓A*β*42 and A*β*40 oligomerization ↓Cell toxicity	Specific binding of retinoic acid to the C-terminal portion of A*β*	[204]
Vitamin D	ROS 17/2.8 cell	10^−6^, 10^−8^, 10^−10^, and 10^−12^ M, 6 h	↑NGF expression	↑AP-1 binding activity in the NGF promoter	[222]
Vitamin E	Rat cortical neurons	1 mM of Trolox (vitamin E derivative) with A*β*	↓A*β*-induced tau phosphorylation	↓P38 MAPK	[231]

**Table 2:** Effects of bioactive compounds on type 2 diabetes mellitus (*in vitro* studies).

Bioactive compounds	Models	Treatment	Effects	Specific mechanism of action	Reference
*Polyphenols*					
Resveratrol	INS-1E, *β* cells, and human islets	25 *μ*M, 24 h	↑Glucose-stimulated insulin secretion ↑Glucose metabolism ↑Mitochondrial activation	↑The activation of SIRT1	[51]
Quercetin	L6 skeletal muscle cells, murine H4IIE cells, human HepG2 hepatocytes	50 *μ*M, 18 h	↑Glucose uptake ↑GLUT4 translocation ↓Hepatic glucose production	↑The activation of AMPK ↓The activity of G6Pase	[61]
Genistein	INS-1 cells, human islets	0.1, 1, and 5 *μ*M 24 h	↑*β*-cell proliferation	↑cAMP/PKA-dependent ERK1/2 signaling pathway	[75]
EGCG	RIN5mF cells	20, 50, 100, and 200 *μ*g/ml, 24 h	↓Cytokine-induced *β*-cell destruction	↓NO ↓iNOS expression through the inhibition of NF-*κ*B activation	[90]
Hesperidin	Pancreatic islets cells	0.2 and 1 mg/ml, 24 h	↑Insulin synthesis and secretion ↑Cell function	↓Oxidative stress induced by IL-1*β*	[109]
Anthocyanins	HepG2 cells	50, 100, and 250 *μ*g/ml, 24 h	↓Insulin resistance ↑Glucose uptake ↑Glycogen content	↑PI3K/Akt pathways ↓G6Pase, PEPCK activity	[121]
Curcumin	STZ-induced islets	10 *μ*M, 24 h	↑Islet viability ↑Insulin secretion	↓ROS, NO ↓Poly ADP-ribose polymerase-1	[131]
Rutin	Rat soleus muscle	10 and 500 *μ*M	↑Glucose uptake	Via the PI3K, atypical protein kinase C and MAPK pathways ↑GLUT4 synthesis	[237]
Naringin	Human umbilical vein endothelial cells	12.5, 25, 50, 100, and 200 *μ*M, 5 days	↓High-glucose-induced damage	↑Mitochondrial function ↓Expression of CX3CL1	[161]

*Vitamins*					
Vitamin A	Fetal and adult rats' pancreatic islets	10^−6^ M retinoic acid, 24 h	↑Insulin mRNA level ↑Insulin secretion	↑Glucokinase through activation of glucokinase promoter	[198]
Vitamin D	Rat RINm5F, human islets	10^−6^ or 10^−8^ M 1,25(OH)_2_D_3_, 48 h	↓Cytokine-induced apoptosis	↑Antiapoptotic A20 gene ↓NF-*κ*B	[215]
Vitamin E	Alloxan-treated mice pancreatic islets	0.01 and 0.1 mM *α*-tocopherol with glucose	↑Insulin secretion	↓Oxidative stress ↓Apoptosis	[54]

**Table 3:** Effects of bioactive compounds on type 2 diabetes mellitus (*in vivo* studies).

Bioactive compounds	Models	Treatment	Effects	Specific mechanism of action	Reference
*Polyphenols*					
Resveratrol	db/db mice	20 mg/kg/day, 12 weeks	↓Glucose tolerance ↓Pancreatic islet fibrosis ↑Islet mass	↓Oxidative stress	[228]
Quercetin	STZ-induced diabetic rats	50 mg/kg/day, orally for 6 weeks	↓Diabetes-induced hypertension and vasoconstriction	↓TNF-*α*, CRP, NF-*κ*B	[47]
Genistein	STZ-induced diabetic rats	250 mg/kg of diet, 6 weeks	↓STZ-induced hyperglycemia ↑Blood insulin level ↑Glucose tolerance	↑*β*-cell proliferation ↓*β*-cell apoptosis	[75]
EGCG	Male db/db mice	250, 500, or 1000 mg/kg of diet, 5 weeks or orally by gavage 30 or 100 mg/kg/d	↑Blood insulin level ↑Glucose tolerance ↓Blood glucose	↑mRNA expression of glucokinase ↓mRNA expression of PEPCK, G6Pase, and fatty acid synthase ↑Pancreatic function	[93]
Hesperidin	HFD/STZ-induced diabetic rats	50 mg/kg/day, orally for 4 weeks	↓HbA1c, glucose level ↑Serum insulin level	↑Antioxidants (vitamin C and vitamin E) and GSH ↓NO, TNF-*α*, and IL-6	[109]
Anthocyanins	STZ-induced diabetic rats	One-time i.p. injection 3 mg/kg bodyweight	↑Blood insulin level ↑Glucose tolerance ↓Blood glucose ↓Oxidative stress	↓Hemoglobin glycation, iron-mediated free radical reactions ↑Hemoglobin-mediated iron release	[122]
Curcumin	STZ-induced diabetic rats	100 mg/kg body weight for 8 weeks	↓Body weight, glucose ↑Blood insulin level ↓Pancreatic *β*-cell damage	↓TNF-*α*, IL1-*β*, and IFN-*γ* ↑Nrf-2, HO-1, and GLUT2 ↓ER/mitochondrial-related apoptosis	[133]
Rutin	S961-treated C57BL/6 mice	Oral gavaged (25 mg/kg body weight) and metformin (100 mg/kg body weight)	↓Blood glucose	↑IRK activity ↑GLUT4 translocation	[147]
Naringin	STZ-induced type 2 diabetic rats	100 mg/kg body weight for 4 weeks	↓Blood glucose ↓Total lipid, triglycerides, and total cholesterol	↑G6Pase activity ↑Insulin receptor, GLUT4, and adiponectin ↓Oxidative stress	[159]
Naringenin	STZ-induced diabetic rats	100 mg/kg body weight for 15 days	↓Blood glucose ↓Total lipid, triglycerides, and LDL and VLDL ↓Oxidative stress	↑Expression of GLUT4 and PPAR*γ*	[172]

*Carotenoid*					
Lycopene	STZ-induced diabetic rats	10, 30, 60, or 90 mg/kg body weight for 30 days	↑Blood insulin level ↓Blood glucose ↓Total lipid, triglycerides, and total cholesterol	↑Activities of antioxidant enzymes ↓NO, H_2_O_2_	[185]

*Vitamins*					
Vitamin A	High-fat/high-sucrose diet-induced obese mouse	Direct pipetting (0.16 mg RA/50 *μ*l in oil) into the mouths	↓Adipose lipid stores ↑Muscle mitochondrial content ↑Glucose tolerance ↓Insulin resistance	↑PPAR*β*/*δ* expression ↑RAR expression	[200]
Vitamin D	Alloxan-induced diabetic rats	1,25(OH)_2_D_3_ intraperitoneal (7 ng/gm/day) for 15 days	↓Pancreatic and liver damage ↓Hyperglycemia	↑DNA tail length of liver and pancreas ↓Serum calcium levels ↓G6Pase, FBPase	[219]
Vitamin E	Alloxan-induced mouse	50 mg *α*-tocopherol, per 100 g diet, 14 weeks	↓Alloxan-induced hyperglycemia ↑Insulin secretion	↓Oxidative stress ↓Pancreas apoptosis	[228]

**Table 4:** Effects of bioactive compounds on Alzheimer's disease (*in vivo* studies).

Bioactive compound	Models	Treatment	Effects	Specific mechanism of action	Reference
*Polyphenols*					
Resveratrol	SAMP8 and SAMR1 mice	Transresveratrol 1 g/kg in diet, 7 months	↑Life expectancy ↓Cognitive impairment in SAMP8 ↓Amyloid deposition	↑AMPK pathways ↑SIRT1 ↑Nonamyloidogenic ADAM-10 enzyme	[239]
Quercetin	3xTg-AD mice	i.p. injection 25 mg/kg every 48 hours for 3 months	↑Learning and memory function	↓A*β*1-40, A*β*1-42, and BACE1 ↓Microglial activation	[70]
Genistein	Intrahippocampal A*β*1-40-injected rats	10 mg/kg, one hour before surgery	↑Short-term spatial recognition memory in a Y-maze test ↑Learning and memory	↓Oxidative stress	[86]
EGCG	APPsw mice	i.p. 20 mg/kg, 60 days, or orally 50 mg/kg, 6 months	↑Memory performance ↓A*β* levels ↓Tau hyperphosphorylation	↓*α*-secretase	[104]
Hesperidin	APP/PS1 mice	Intragastric administration 40 mg/kg for 90 days	↑Learning and memory function	↓Oxidative stress via activation of Akt/Nrf2 ↓Inflammation via inhibition of RAGE/NF-*κ*B	[116]
Anthocyanins	APPsw mice	ANT-rich blackcurrant extracts 5.6 mg/day, 6 weeks	↑Spatial working memory	↓Oxidative stress	[127]
Curcumin	Alzheimer transgenic APPsw mouse model	Low dose: 160 ppm or high dose: 5000 ppm, 6 months	↓Overall insoluble and soluble amyloid, and plaque burden (low dose) ↓Oxidative stress and inflammation	↓IL1-*β*, IL-6, and ApoE ↓NF-*κ*B, iNOS, and COX-2 ↓Plasma and tissue cholesterol	[139]
Rutin	A*β*1-42-injected rats	100 mg/kg body weight/day, 3 weeks	↓A*β*-induced learning and memory deficits ↓A*β*-induced neurotoxicity	↑Activation of MAPK pathway ↑BDNF gene expression	[156]
Naringin	APPswe/PS1dE9 transgenic mouse	50 or 100 mg/kg body weight/day, 16 weeks	↑Learning and memory ability	↑CaMKII activity	[167]
Naringenin	A*β*1-40-injected Wistar rats	Orally by gavage at a dose of 100 mg/kg one hour before surgery	↓A*β*-induced learning and memory deficits	↓Lipid peroxidation ↓Apoptosis estrogenic pathway	[179]

*Carotenoid*					
Lycopene	A*β*1-42-injected Wistar rats	1, 2, and 4 mg/kg, orally 14 days	↓A*β*-induced learning and memory deficits	↓NF-*κ*B, TNF-*α*, and IL-1*β*	[193]

*Vitamins*					
Vitamin A	APP/PS1 mice	i.p. 20 mg/kg all-trans-retinoic acid, 3 times/week, 8 weeks	↓Spatial learning and memory ↓A*β* accumulation ↓Tau hyperphosphorylation	↓APP processing ↓CDK5 activity ↓Activated microglia and astrocytes	[207]
Vitamin D	APP/PS1 mice	0 (deficiency diet), 2.4 (control diet), and 12 IU/g (surplus diet), 5 months	↓Amyloid plaques ↓A*β* peptides	↓Neuroinflammation ↑NGF	[240]
Vitamin E	Tg2576 mice	8 IU/g/day, 6 months	↓A*β* peptide formation in young but not in old Tg2576 mice	↓Oxidative stress	[241]
